# Grain and Bean Lysates Improve Function of Endothelial Progenitor Cells from Human Peripheral Blood: Involvement of the Endogenous Antioxidant Defenses

**DOI:** 10.1371/journal.pone.0109298

**Published:** 2014-10-17

**Authors:** Daniela Lucchesi, Rossella Russo, Morena Gabriele, Vincenzo Longo, Stefano Del Prato, Giuseppe Penno, Laura Pucci

**Affiliations:** 1 Department of Clinical and Experimental Medicine, Section of Metabolic Diseases, University of Pisa, Pisa, Italy; 2 Institute of Agricultural Biology and Biotechnology, National Research Council, CNR, Pisa, Italy; Scuola Superiore Sant'Anna, Italy

## Abstract

Increased oxidative stress contributes to the functional impairment of endothelial progenitor cells (EPCs), the pivotal players in the servicing of the endothelial cell lining. Several evidences suggest that decreasing oxidative stress by natural compounds with antioxidant properties may improve EPCs bioactivity. Here, we investigated the effects of Lisosan G (LG), a *Triticum Sativum* grain powder, and Lady Joy (LJ), a bean lysate, on function of EPCs exposed to oxidative stress. Peripheral blood mononuclear cells were isolated and plated on fibronectin-coated culture dishes; adherent cells, identified as early EPCs, were pre-treated with different concentrations of LG and LJ and incubated with hydrogen peroxide (H_2_O_2_). Viability, senescence, adhesion, ROS production and antioxidant enzymes gene expression were evaluated. Lysate-mediated Nrf-2 (nuclear factor (erythroid-derived 2)-like 2)/ARE (antioxidant response element) activation, a modulator of oxidative stress, was assessed by immunocytochemistry. Lady Joy 0.35–0.7 mg/ml increases EPCs viability; pre-treatment with either LG 0.7 mg/ml and LJ 0.35–0.7 mg/ml protect EPCs viability against H_2_O_2_-induced injury. LG 0.7 and LJ 0.35–0.7 mg/ml improve EPCs adhesion; pre-treatment with either LG 0.35 and 0.7 mg/ml or LJ 0.35, 0.7 and 1.4 mg/ml preserve adhesiveness of EPCs exposed to H_2_O_2_. Senescence is attenuated in EPCs incubated with lysates 0.35 mg/ml. After exposure to H_2_O_2_, LG pre-treated cells show a lower senescence than untreated EPCs. Lysates significantly decrease H_2_O_2_-induced ROS generation. Both lysates increase glutathione peroxidase-1 and superoxide dismutase-2 (SOD-2) expression; upon H_2_O_2_ exposure, pre-treatment with LJ allows higher SOD-2 expression. Heme oxigenase-1 increases in EPCs pre-treated with LG even upon H_2_O_2_ exposure. Finally, incubation with LG 0.7 mg/ml results in Nrf-2 translocation into the nucleus both at baseline and after the oxidative challenge. Our data suggest a protective effect of lysates on EPCs exposed to oxidative stress through the involvement of antioxidant systems. Lisosan G seems to activate the Nrf-2/ARE pathways.

## Introduction

Bioactive compounds in food might have beneficial effects on human health relieving complex disorders such as obesity, type 2 diabetes, dyslipoproteinaemias, hypertension, heart and vascular diseases and cancer. Recent studies indicate a very high potential of tailored nutritional intervention strategies in preventing all these disorders [1]. The characterization of bioactive compounds of staple foods represents an essential step in the process of validation of such nutritional strategies and is central for specific “functional” food definition [2].

“Nutraceutical”, a combination of nutrition and pharmaceutical, indicates a food component that provides health benefits, including the prevention of diseases. Different experimental approaches are commonly used for the assessment ot the nutraceutical value of foodstuff and ingredients. In particular, the use of *in vitro* cell models represents a powerful and informative tool for the definition of their antioxidant capacity and related cytoprotective effects [3].

Bone marrow (BM)-derived endothelial progenitor cells (EPCs) play an important role in endothelium maintenance by means of reparatory mechanisms such as re-endothelialization and neoangiogenesis. Representing an emerging actor directly involved in vascular competence, EPCs offer a cell model of great interest [4], [5]. All atherosclerotic risk factors adversely affect circulating EPCs number, functional properties and senescence [6], [7]. These are prominent contributors to the onset and progression of cardiovascular disease in many clinical conditions [8].

Recent technological advances have challenged and redefined several aspects of the biology and function of EPCs. First of all, the contribution to endothelial repair and angiogenesis made by these BM-derived cells by integrating into the endothelial layer seems smaller than traditionally believed [9]. Furthermore, methods commonly (and herein) used for isolating EPCs in short-term cultures generate a mixed population of myeloid cells, also termed early EPCs, which are not truly endothelial precursors even if they assume and endothelial-like phenotype by virtue of the expression of endothelial markers such as vascular endothelial growth factor receptor-2 (VEGF-R2), CD31 and von Willebrand factor (vWf). Nonetheless, these cells, mainly representative of the hematopoietic lineage rather than EPCs, though not able to give rise directly to patent vessels in vivo as well as tubular structures in vitro, can contribute to vascular repair depending on their ability to release in a paracrine manner several growth factors and cytokines [10], [11].

Early EPCs have already been studied in the nutraceutical field; examples of food-derived bioactive compounds with beneficial effect on function and number of early-EPCs include resveratrol and Ginkgo biloba extract.

Resveratrol is likely to contribute to the potential of red wine to prevent cardiovascular disease by increasing nitric oxide (NO) bioavailability that enhances number and function of circulating EPCs. Wang *et al.* [12] showed that resveratrol delays EPCs senescence by increasing telomerase activity. Consistently, Ginkgo biloba extract reduces in a dose and time dependent manner EPCs senescence by activating telomerase through the PI3k/Akt signaling pathway [13]. In patients with type 1 diabetes, folic acid normalizes EPCs gene expression profile suggesting that signaling pathways modulated by folates may be therapeutic targets to improve EPCs function [14]. Circulating EPCs increased by more than one fold in patients with cardiovascular disease who had higher intake of isoflavone (a major component of phytoestrogen), suggesting that this compound may confer vascular protection through enhanced endothelial repair [15]. Conversely, 12 weeks of fish-oil supplemention had no beneficial effect on vascular endothelial function and EPCs count, with no changes in metabolic profiles, inflammation or oxidative stress in patients with type 2 diabetes [16]. These evidences suggest controversial effects of natural compounds on EPCs number and function.

Epidemiological studies correlate the intake of whole grain and whole-grain products with a reduced incidence of cardiovascular disease, diabetes and cancer [17], [18], [19], [20], [21], [22]. The involvement of reactive oxygen species (ROS) in the aetiology of these degenerative conditions has suggested that whole grain phytochemicals with antioxidant activity may contribute to these benefits on human health [23].

Lisosan G (LG) is a dry powder of grain obtained from *Triticum Sativum* and registered as a nutritional integrator: it contains vitamin B, tocopherols and polyunsaturated fatty acids. LG does not interfere with hepatic drug-metabolizing enzymes and protects rats against both carbon tetrachloride- and cisplatin-induced toxicity through radical scavenging, attenuation of oxidative stress and saving of antioxidant enzymes [24], [25]. Lady Joy is a variety of bean viable as a source of phaseolamin, not contaminated by toxic phytohemagglutinin [26]. As an inhibitor of alpha-amylase with a direct negative effect on the digestion of starch, phaseolamin has been proposed as a remedy against obesity and diabetes [27], [28]. From this variety of bean the Lady Joy (LJ) lysate has been prepared.

To counteract ROS-induced damage, several genes encoding detoxifying and antioxidant proteins are expressed in human cells [29], [30]. This response is regulated through a *cis*-acting element, antioxidant responsive element (ARE) or electrophile responsive element (EpRE), within the regulatory region of target genes [31], [32]. Nrf-2, nuclear factor (erythroid-derived 2)-like 2 [33], has been recently identified as the major regulator of ARE-mediated gene expression [34]; this factor regulates the expression of several genes encoding drug metabolizing enzymes and anti-oxidant proteins, including heme oxigenase-1 (HO-1) and superoxide dismutase-2 (SOD2) [35]. Recent data show that dietary polyphenols are able to induce detoxifying and antioxidant defenses through ARE/Nrf-2 pathway activation; for instance, resveratrol increases cellular antioxidants and phase II enzymes activity by Nrf-2 induction [36].

Here, we have first evaluated total content of polyphenols and flavonoids, a class of polyphenols, in Lisosan G and Lady Joy lysates; then we have investigated the effects of LG and LJ on the functional properties of EPCs in basal conditions and upon exposure to oxidative stress injury induced by hydrogen peroxide (H_2_O_2_). Furthermore, we have assessed the expression of several enzymes representing the endogenous intracellular antioxidant defense system. Finally, the role of Nrf-2, a transcription factor that functions as the key controller of the redox homeostatic regulatory network, has been preliminary explored.

## Materials and Methods

### Ethics Statement

Blood samples were obtained from healthy volunteers under written consent according to the Declaration of Helsinki, and protocol was approved by local Ethics Committee.

### Study Protocol

To evaluate the effect of Lisosan G (supplied by Agrisan Company, Pistoia, Italy) and Lady Joy lysates (supplied by Institute of Agricultural Biology and Biotechnology, IBBA, CNR Milano, Italy) on endothelial progenitor cells bioactivity, early EPCs were incubated for 4 hours at increasing doses of each compound (0, 0.35, 0.70 and 1.4 mg/ml); further, in a new set of experiments, each compound was added to the medium before the treatment with 1 mM hydrogen peroxide (H_2_O_2_).

### Cell culture

Following overnight fasting, venous blood from healthy volunteers was drawn in EDTA tubes and processed within 2 hours from collection. Peripheral blood mononuclear cells (PBMCs) were fractionated using Biocoll density-gradient centrifugation (Biochrom AG; density = 1.077 g/ml). PBMCs (1×10^6^ cells/cm^2^) were seeded on 2 µg/cm^2^ fibronectin coated culture dishes (BD Falcon) or Lab-Tek II chamber slides system (Sigma-Aldrich Ltd, Poole, Dorset, UK) after red cell lysis. Cells were cultured in endothelial basal medium (EBM-2, Lonza Sales AG, Basel, Switzerland) supplemented with EGM-2-MV-SingleQuots containing human endothelial growth factor, hydrocortisone, insulin-like growth factor, fibroblast growth factor, vascular endothelial growth factor (VEGF), antibiotics and 5% fetal bovine serum (FBS, Lonza Sales AG). After 3 days culture, non-adherent cells were discarded by washing with PBS and the culture medium replenished daily. On day 5, adherent cells, displaying an elongated spindle-shaped morphology, were identified as early EPCs.

### EPCs characterization

Early EPCs were characterized for the uptake of 1,1′-dioctadecyl-3,3,3′,3′-tetramethylindocarbocyanine-labeled acetylated Low-Density Lipoprotein (DiI-Ac-LDL) and lectin binding. The staining was performed by incubating EPCs with 10 µg/ml of DiI-Ac-LDL (Invitrogen, Life Technologies Ltd, Paisley, UK) for 2 hours at 37°C. Cells were fixed in 4% paraformaldehyde for 30 min and counterstained with 1 mg/ml FITC-labelled lectin from *Ulex europaeus* (Sigma-Aldrich Ltd) for 2 hours at 37°C in dark. Images of the stained cells were viewed with a fluorescence microscope and double positive DiI-Ac-LDL/Lectin cells were preliminary identified as early EPCs. As previously described [37], cells were further characterized by demonstrating the expression of CD31, vWf, KDR, VE-chaderin and CD14 by flowcytometry (data not shown).

### Total polyphenol and flavonoid contents

To estimate total polyphenols concentration we used a modified Folin-Ciocalteu assay. A standard curve was generated using gallic acid [38], [39]. The powders of LG and LJ lysate were sonicated in water (three cycles: 10s on/10s off); after centrifugation, Folin-Ciocalteu reagent was added to supernatants and to different concentrations of gallic acid. After 8 min incubation, a solution of sodium carbonate 0.7 M was added for 2 hours in dark, followed by measurement of the optical density at 750 nm. Results are expressed as mg of gallic acid equivalent (GAE) per 1 g of dry weight (dw) powder (mg GAE/g dw powder).

Total flavonoid content was determined according to the colorimetric methods described by Lee *et al*. [40]. Briefly, appropriate dilutions of sample extracts were reacted first with sodium nitrite, then with aluminium chloride to form a flavonoid-aluminium complex. Solution absorbance at 430 nm was immediately measured and compared to quercetin standards. Flavonoid content was expressed as mg of quercetin equivalent (QE) per 1 g of powder (mg QE/g dw powder).

Finally, we evaluated total antioxidant activity of both lysates by oxigen radical absorbance capacity (ORAC) method. ORAC was measured accordingly to the procedure by Huang *et al.* [41] and expressed as µmol trolox equivalent (TE) for 1 g of powder (µmol TE/g dw powder).

### Assessment of cell viability

The MTT assay was performed to evaluate viability of cultured EPCs. MTT (3-(4,5-dimethylthiazol-2-yl)-2,5-diphenyltetrazolium bromide) measures mitochondrial activity in living cells. Briefly, after 5–7 days of culture, EPCs were incubated with MTT (Sigma, St. Louis, MO, USA) (1 mg/mL) for 3 hours at 37°C, 5% CO_2_. Upon incubation, the medium was removed and the cells solubilized in 10%DMSO/90%Isopropanol. Then, the amount of the dye released from the cells was quantified by measuring the optical density at 540 nm (reference wavelength: 620 nm) by use of a multiplate reader (Multiskan EX, THERMO). The optical density is directly correlated with the amount of metabolically active cells. Since the MTT assay does not readily discriminate between an increase in cell proliferation and an higher resistance to apoptosis, we assessed whether both lysates stimulate EPCs proliferation. To this purpose, cells have been expanded by adding several concentrations of lysates to the complete EGM-2 medium. The Trypan Blue staining, a dye exclusion procedure, was employed for viable cells counting.

### EPC adhesion assay

After being treated with lysates for 4 hours, EPCs were washed with PBS, and then gently detached with 0.25% trypsin/EDTA. After centrifugation and re-suspension, equal cell numbers were seeded on fibronectin coated culture dishes, and incubated for 30 min at 37°C in a medium free of EGM-2. Adherent cells were counted in five random high-power (x 200) microscope fields (HPF)/well by two independent observers unaware of the treatments.

### Senescence

Senescent cells were identified using the Senescence Cells Histochemical Staining kit (Sigma). Briefly, EPCs were washed in PBS, fixed for 7 minutes at room temperature, washed again and incubated for 16–18 hours at 37°C (no CO_2_) with X-gal chromogenic substrate. The cells were then washed with PBS, added with DMSO for dissolving the stain, and incubated at 37°C for 30 min, followed by measurement of absorbance at 620 nm.

### ROS production

ROS production was evaluated by ROS-sensitive fluorescent probe 5-(and-6)-chloromethyl-2′,7′-dichloro-di-hydro-fluorescein diacetate, acetyl ester (CM-H_2_DCFDA) (Invitrogen, Life Technologies Ltd). Briefly, EPCs were incubated with CM-H_2_DCFDA (10 µM/well) for 30 min at room temperature in the dark and ROS production was detected by measuring the increase in fluorescence, by a microplate reader. Fluorescence was measured by excitation at 495 nm and emission at 527 nm.

### RT-PCR and TaqMan real-time PCR

EPCs were trated with LG 0.7 mg/ml and LJ 0.35 mg/ml. RNA was extracted using TRIzol reagent (Gibco, Life Technologies Inc., Carlsbad, CA, USA). The quantity and purity of the isolated RNA were measured by optical density; the extracted RNA showed an OD_280/260_ ratio between 1.8 and 2.0. For RNA purification we used deoxyribonuclease I (Invitrogen, Life Technologies Ltd) which digests single and double stranded DNA to oligodeoxy-ribonucleotides containing a 5-phosphate. One microgram of total RNA from each sample was reverse transcribed to cDNA with an iScript cDNA Synthesis kit (Bio-Rad, Richmond, CA, USA) according to manufacturer's protocol. Real-time quantitative PCR was carried out using the Applied Biosystem Step One Plus (PE Applied Biosystem, Warrington, UK). For gene expression analysis of catalase (CAT), superoxide dismutase 2 (SOD2), glutathione peroxidase type 1 (GPx-1) and heme oxygenase-1 (HO-1) the pre-developed TaqMan gene expression assays (PE Applied Biosystems) and the TaqMan Universal PCR Master Mix (PE Applied Biosystems) have been employed. We quantified gene expression using a comparative critical threshold (*C*_t_) method; *C*_t_ numbers were used to calculate the expression levels of genes normalized to housekeeping, which consists of endogenous cellular 18S rRNA (PE Applied Biosystems). All samples were assayed in triplicate and means were presented as fold-increase compared to control.

### Activation of Nrf-2

We explored the role of transcription factor Nrf-2 [Nuclear factor (erythroid-derived 2)-like 2] which promotes mRNA expression and activity of antioxidant enzymes. Translocation of Nrf-2 to the nucleus was evaluated by fluorescence microscopy. Briefly, EPCs were washed in PBS, fixed with 4% (wt/vol) formaldehyde in PBS for 30 min at room temperature. After washing with PBS, the chamber slides were incubated with 0.2% TRITON X-100 in PBS for 10 min and then blocked with 1% BSA in PBS for 1 hour, followed by overnight incubation with anti-Nrf-2 (H-300) (Santa Cruz Biotechnology, Santa Cruz, CA, USA) at room temperature. The chamber slides were washed extensively before incubation for 1 hour with a secondary mouse anti-rabbit IgG-FITC antibody (Santa Cruz Biotechnology) in dark. The cells were washed with PBS and viewed with a fluorescence microscope. We used DAPI as nuclei-specific dye.

### Statistical analysis

Statistical analysis were carried out using SPSS 13.0 software (SPSS Inc., Chicago, I, USA) for Mac OS X. All data are expressed as means ± SE of at least 3 independent experiments. Unpaired Student’s t-test (two-tailed) was used for single comparisons, while one-way ANOVA with Fisher’s Least Significant Difference (LSD) post-hoc test was carried out for multiple comparisons. *P* values<0.05 were considered statistically significant.

## Results

### Total polyphenol and flavonoid contents of lysates

LG lysate showed a slightly higher level of polyphenols content than LJ lysate (5.209±0.298 vs 2.290±0.050 mg GAE/g dw powder; means±SE), while flavonoids concentration was higher in LJ than LG lysate (0.281±0.006 vs 0.110±0.005 mg QE/g dw powder). Total polyphenol and flavonoid content of lysates did not change significantly after increasing sonication time and cycles number of powders treatment. Consistently, ORAC was higher in LG as compared with LJ (16.77±0.84 vs 7.43±0.353 µmol TE/g dw powder), and there was a significant linear correlation between the concentration of total polyphenols and ORAC in the investigated powders (r = 0.963).

### EPCs viability

**Figure 1:**
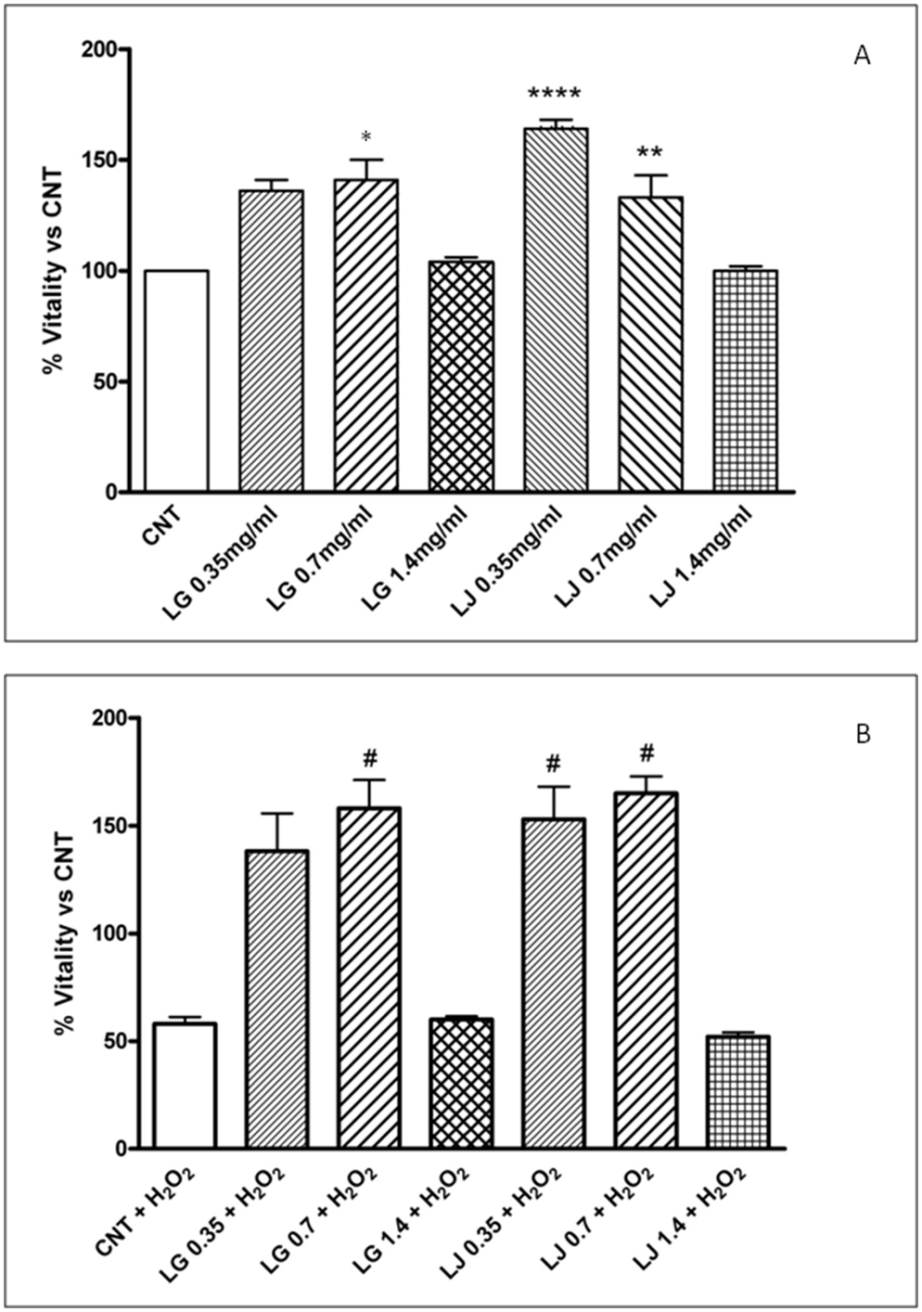
Effects of Lisosan G (LG) and LJ lysate (LJ) on viability of EPCs in absence (panel A) or presence of oxidative stress induced by H_2_O_2_ (panel B). Data are expressed as means ±SE; n≥3. *p<0.05, **p<0.01, ****p<0.001 vs control (CNT); #p<0.05 vs CNT + H_2_O_2_.

EPCs were treated with different doses of LG and LJ lysates (0.35, 0.7 and 1.4 mg/ml) for 4 hours. Compared to control cells (CNT), EPCs viability tested by MTT significantly improved after treatment with LJ at 0.35 mg/ml (p<0.001) and after exposure to both lysates at 0.7 mg/ml (p<0.01 for LJ; p<0.05 for LG). The highest doses of both lysates (1.4 mg/ml) showed no effect on cell viability (Figure 1, panel A). Cell counting by Trypan Blue somehow mimics results obtained with the MTT assay, but the number of viable cells per ml of culture resulted significantly increased (p<0.05) only for LJ 0.35 mg/ml, not for LG and LJ at 0.7 mg/ml (data not shown). Thus, the increased metabolic activity demonstrated by MTT seems not related to an increased number of viable cells.

Exposure to H_2_O_2_ (CNT + H_2_O_2_) halved EPCs viability of cells untreated with lysates (p<0.01 vs CNT). Pre-treatment with LG 0.7 mg/ml (p<0.05) and LJ 0.35 and 0.7 mg/ml (p<0.05 for both) protected cells by H_2_O_2_-induced toxicity (Figure 1, panel B). Both LG and LJ at 1.4 mg/ml did not preserve viability of EPCs exposed to H_2_O_2_ that was superimposable to the viability of the H_2_O_2_-treated control cells (Figure 1, panel B).

### EPCs adhesion capacity

**Figure 2:**
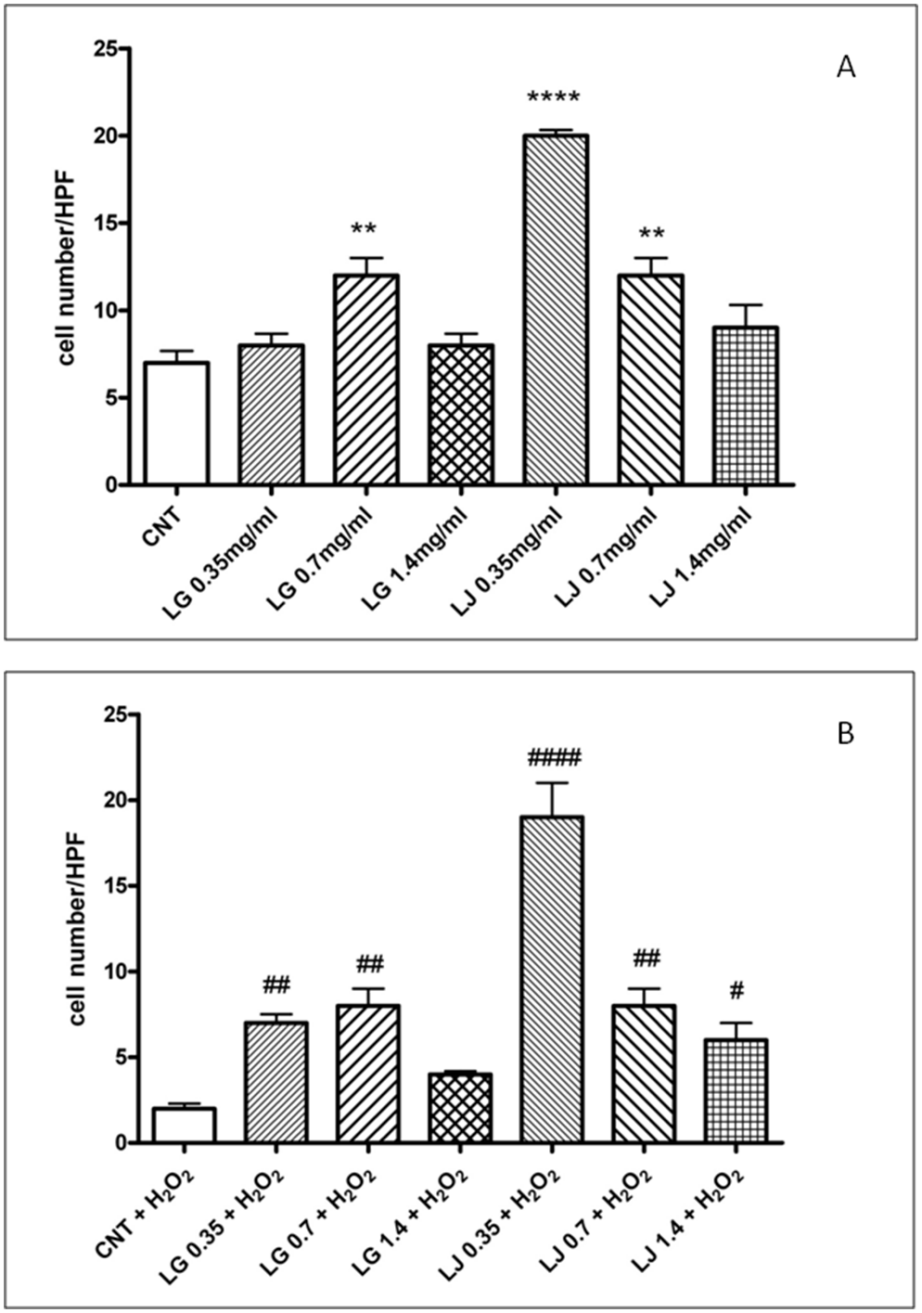
Effects of Lisosan G (LG) and LJ lysate (LJ) on EPCs adhesion in absence (panel A) or presence of oxidative stress induced by H_2_O_2_ (panel B). Data are expressed as means ±SE; n≥3. **p<0.01, ****p<0.001 vs control (CNT); # p<0.05, ## p<0.01, #### p<0.001 vs CNT + H_2_O_2_.

Compared to CNT, pre-treatment with LG 0.7 mg/ml (p<0.01) and both LJ 0.35 (p<0.001) and 0.7 mg/ml (p<0.01) improved EPCs adhesion, while pre-treatment with LG and LJ at 1.4 mg/ml did not affect this property (Figure 2, panel A).

The exposure to 1 mM H_2_O_2_ decreased adhesion in untreated EPCs (p<0.01 compared to CNT; Figure 2, panel B). Pre-treatment with LG at both 0.35 and 0.7 mg/ml (p<0.01) and with LJ at 0.7 mg/ml (p<0.01), 1.4 mg/ml (p<0.05) and to a larger extent at 0.35 mg/ml (p<0.001) restored almost completely cell adhesion of EPCs exposed to H_2_O_2_ (Figure 2, panel B).

### EPCs senescence

**Figure 3:**
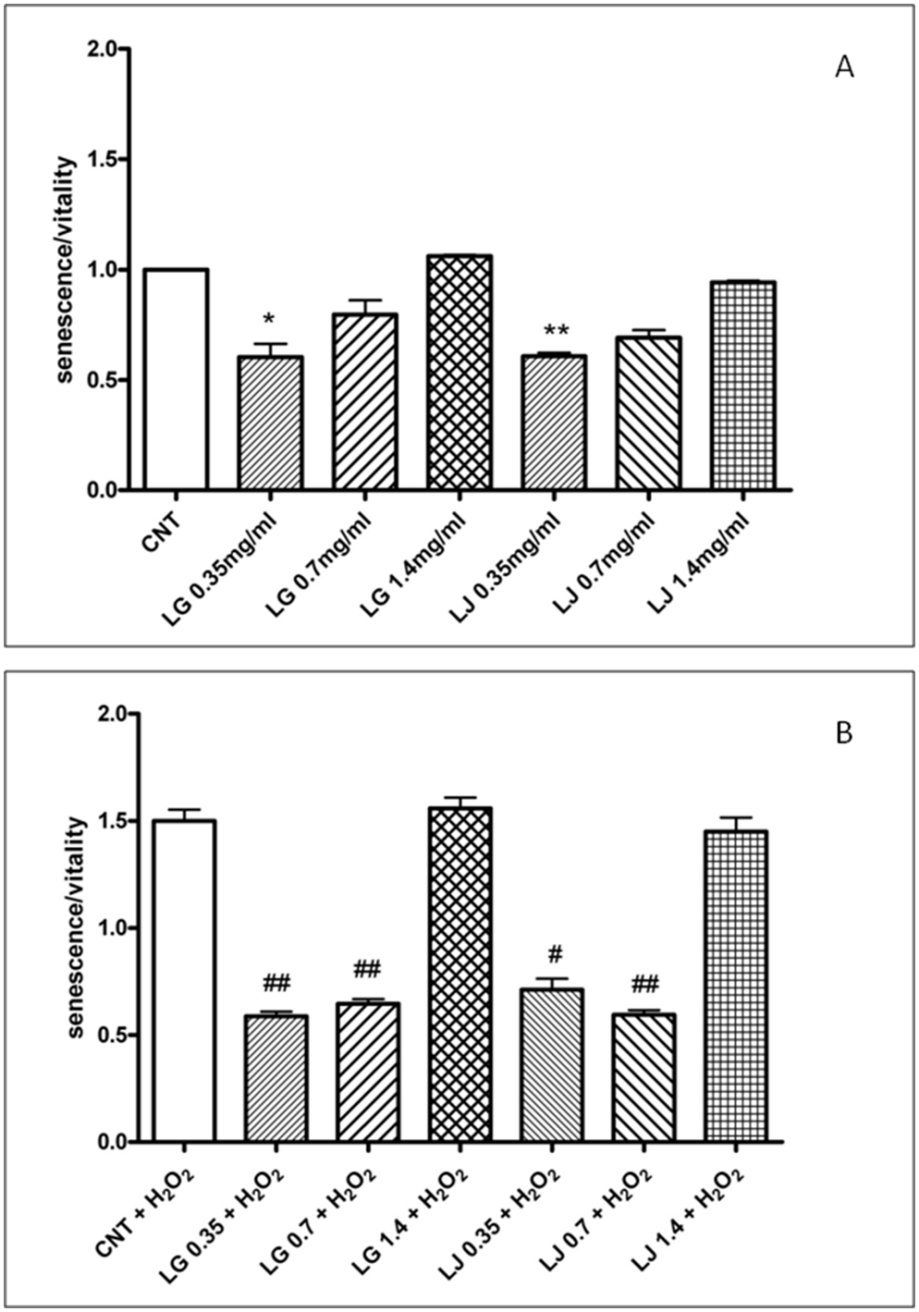
Effects of Lisosan G (LG) and LJ lysate (LJ) on EPCs senescence in absence (panel A) or presence of oxidative stress induced by H_2_O_2_ (panel B). Data are expressed as means ±SE; n≥3. *p<0.05, **p<0.01 vs control (CNT); # p<0.05, ## p<0.01 vs CNT + H_2_O_2_.

EPCs senescence (normalized for viability) was unaffected by both LG and LJ at 0.7 and 1.4 mg/ml and slightly reduced by both LG and LJ at 0.35 mg/ml compared to CNT, (p<0.05 and p<0.01, respectively) (Figure 3, panel A).

Exposure to 1 mM H_2_O_2_ significantly increased senescence of untreated EPCs (p<0.01 compared with CNT; Figure 3, panel B). Pre-treatment with LG at 0.35 and 0.7 mg/ml (p<0.01 for both) and LJ at 0.35 mg/ml (p<0.05) and 0.7 mg/ml (p<0.01) significantly attenuated senescence of EPCs exposed to H_2_O_2_ (Figure 3, panel B). Finally, pre-treatment with both LG and LJ at 1.4 mg/ml did not affect senescence of EPCs exposed to H_2_O_2_ (Figure 3, panel B).

### ROS production

**Figure 4:**
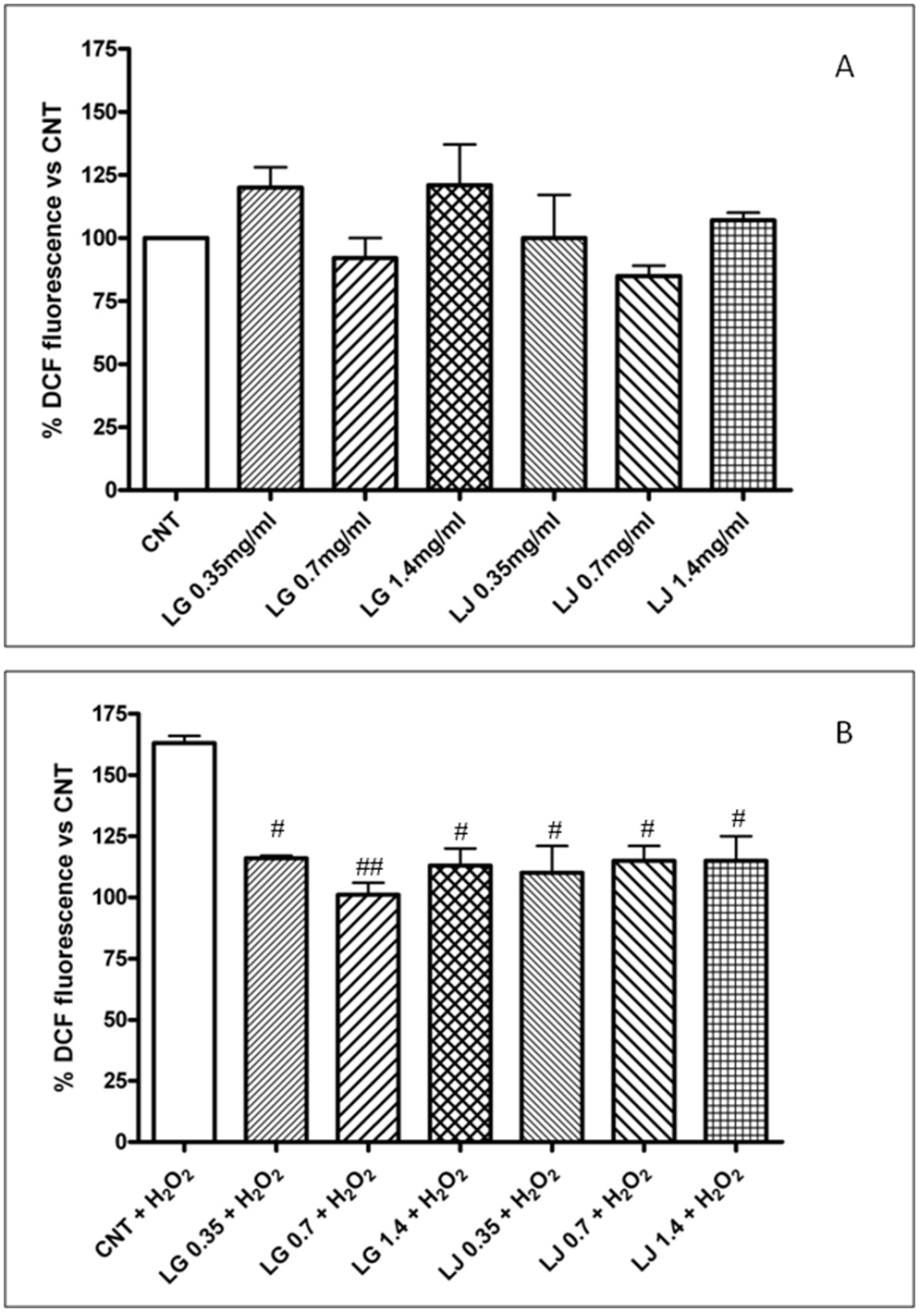
Effects of LG and LJ on ROS production in absence (panel A) or in presence of oxidative stress induced by H_2_O_2_ (panel B). Data are expressed as means ± SE; n≥3. # p<0.05, ## p<0.01 vs CNT + H_2_O_2_.

Compared to CNT, pre-treatment with both lysates at 0.35, 0.7 and 1.4 mg/ml, in the absence of oxidative stress induced by H_2_O_2_, did not change intracellular ROS production (Figure 4, panel A). ROS generation, as determined by CM-H2DCFDA, a cell-permeable indicator for these compounds, increased significantly in EPCs exposed to H_2_O_2_ (p<0.05 compared to CNT) (Figure 4, panel B). Pre-treatment with both lysates normalizes ROS production in EPCs exposed to H_2_O_2_. Indeed, ROS generation decreased significantly in EPCs exposed to H_2_O_2_ and pre-treated with both LG and LJ at all the concentrations tested (p<0.05 versus CNT + H_2_O_2_), with LG 0.7 mg/ml showing an higher efficacy (p<0.01 vs CNT + H_2_O_2_) (Figure 4, panel B).

### Antioxidant enzymes gene expression

**Figure 5:**
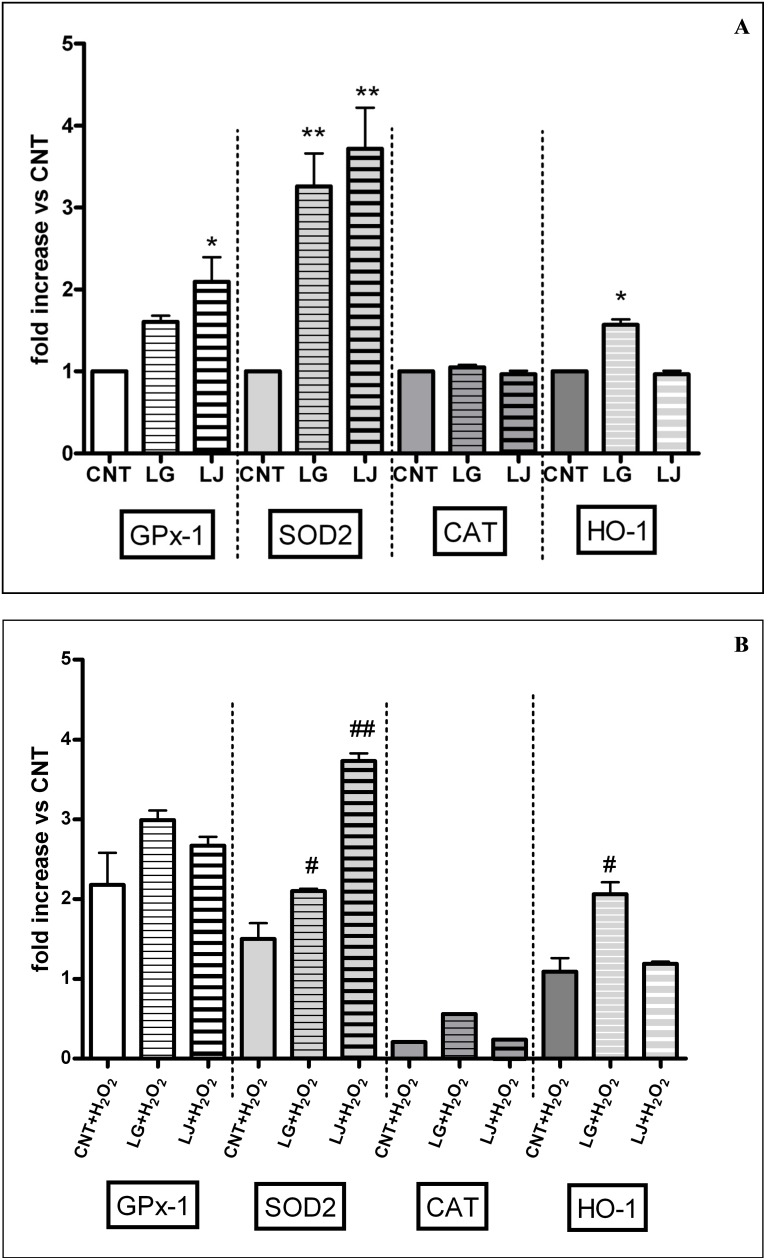
Quantitative real-time RT-PCR analysis of GPx-1, SOD2, CAT and HO-1 expression in EPCs after incubation with lysates in absence (panel A) or in presence of oxidative stress induced by H_2_O_2_ (panel B). The bars represent mean±SE fold increase in transcript expression relative to untreated cells (CNT); n≥3. *p<0.05, **p<0.01 vs control (CNT); # p<0.05, ## p<0.01 vs CNT + H_2_O_2_.

Respect to controls, GPx-1 expression was higher in EPCs pre-treated with LG 0.7 mg/ml and LJ 0.35 mg/ml (p<0.05 for both). Also SOD2 expression was affected by lysates: pre-treatment with LG 0.7 mg/ml and LJ 0.35 mg/ml induce a significant increase in SOD2 expression (p<0.01 vs CNT for both). HO-1 expression increased only in EPCs pre-treated with LG 0.7 (p<0.05), while no effects were observed for both lysates on CAT expression (Figure 5, panel A).

In EPCs untreated with lysates, exposure to H_2_O_2_ increased by two fold and 1.5 fold the expression of GPx-1 and SOD2, respectively (p<0.05 vs CNT for both), while significantly reduced the expression of CAT (p<0.05 vs CNT) and had no effect on HO-1 expression (Figure 5, panel B). Pre-treatment with lysates of EPCs exposed to H_2_O_2_ did not affect GPx-1 and CAT expression, while increased SOD2 expression (to a larger extent LJ 0.35 mg/ml, p<0.01 vs CNT + H_2_O_2_); finally, only LG 0.7 mg/ml significantly increased HO-1 expression (p<0.05) (Figure 5, panel B).

### Activation of Nrf-2

**Figure 6:**
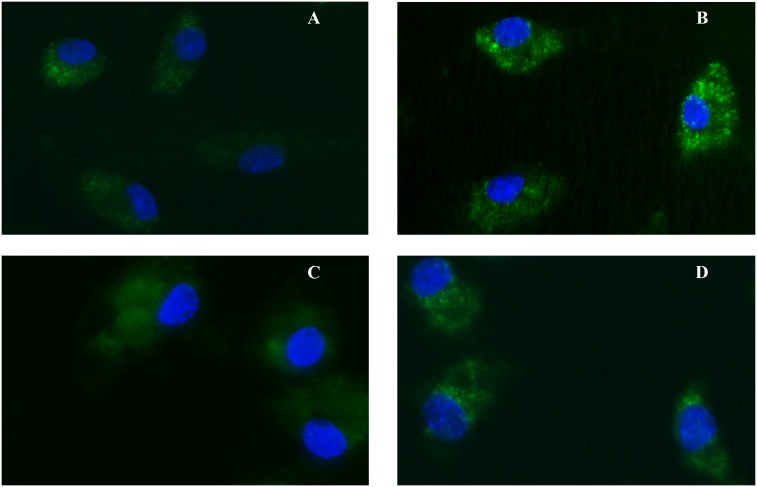
Assessment of Nrf-2 translocation into the nucleus by fluorescence microscopy. Untreated EPCs (panel A); untreated EPCs exposed to oxidative stress induced by H_2_O_2_ (panel C). Nucleus localization of Nrf-2 (green) was present in both EPCs pre-treated with LG (panel B), and also in EPCs exposed to oxidative stress induced by H_2_O_2_ and pre-treated with LG (panel D). DAPI was used for nuclei staining (blue).

To evaluate the activation of Nrf-2, the EPCs were exposed to lysates for 1.5-, 3- and 4-hour periods and immunocytochemistry of this nuclear factor was performed. EPCs pre-treatment with LG 0.7 mg/ml for 3 hours resulted in Nrf-2 translocation from the cytoplasm into the nucleus both in basal conditions (Figure 6, panel B) as well as in presence of the H_2_O_2_-induced oxidative insult (Figure 6, panel D); however, after 4 hours, Nrf-2 was localized in the cytosol again and no more into the nucleus. Exposure to H_2_O_2_, per se, is not able to activate Nrf-2 translocation into the nucleus in untreated cells (Figure 6, panel C). Furthermore, Nrf-2 translocation became apparent, thought weakly, after LJ 0.35 mg/ml pre-treatment for 4 hours (data not shown).

## Discussion

Endothelial progenitor cells participate to vascular homeostasis and are actively involved in maintaining an intact and functional endothelium [42]. EPCs are very rare in the peripheral circulation and are further reduced in subjects with cardiovascular diseases. Moreover, decreased number and impaired function of EPCs may contribute to endothelial dysfunction and susceptibility to cardiovascular events. It has become increasingly evident that these changes in EPCs may be due to enhanced oxidative stress, possibly as a result of systemic or localized inflammation [43]. ROS exert direct cytotoxic effects on the vascular endothelium and on EPCs. Indeed, conditions associated with increased generation of ROS are characterized by reduced circulating EPCs levels and lead to the release of functionally defective EPCs.

Recently EPCs number and function have become the target of pharmaceutical compounds such as statins, renin-angiotensin system blockers, and some classes of glucose-lowering agents (thiazolidinediones and dipeptidyl peptidase-4 inhibitors) [44], [45]; furthermore, several natural anti-oxidative compounds with anti-inflammatory properties have also been found to enhance EPCs bioactivity, even if large clinical trials testing the effects of antioxidants on cardiovascular risk resulted in systematic failures [46], [47]. These disappointing results are probably related to the limited effects of traditional antioxidants on intracellular ROS production, while they exert a scavenging action on already formed ROS [48].

Nevertheless, several antioxidant agents identified among nutraceuticals have been reported to restore EPCs bioactivity. Among them there are puerarin [49], resveratrol/red wine [12], [50], *Ginkgo biloba* [13], berberine [51], salvianolic acids [52], ginsenoside [53], fish oil (rich in long-chain n-3 polyunsaturated fatty acids, PUFAs) [54], and salidroside (the major phenylpropanoid glycoside derived from Rhodiola) [55].

For the first time, the present study demonstrated that grain and bean lysates, LG and LJ respectively, enhance viability and adhesion capacity of EPCs while reducing their senescence. Overall, in basal conditions, i.e. with no exposure to oxidative stress, both lysates promote viability and adhesiveness and reduce senescence at low (0.35 mg/ml) and medium concentrations (0.7 mg/ml), but not at the highest levels (1.4 mg/ml). Thus, it appears that both lysates do not affect EPCs functional activities in a dose dependent manner, while the largest effects of LJ on viability and adhesion capacity occur at the lowest concentrations (Figures 1–3, panels A). A double-edged or biphasic action on EPCs with no or detrimental effects at higher-doses has been reported for several compounds including statins, thiazolidinediones and even insulin [44], [56]. Also resveratrol, at low doses promotes antioxidant effects while at higher doses can have a dose-dependent pro-oxidant role followed by cell damage and apoptosis [57]. Based on the aforementioned results, it is possible to speculate that, through improvement in viability, adhesiveness and senescence, both lysates at low concentrations could improve EPCs performance in the maintenance of the endothelium homeostasis.

It is interesting to observe that under the conditions used in the first step of the present study (no exposure to oxidative stress) all these effects occur with no changes in ROS production (Figure 4, panel A). On the other hand, compared to control EPCs, expression of GPx-1 increased in cells treated with both LG and LJ, expression of HO-1 increased only in cells treated with LG, while expression of SOD2 was largely increased by both, with no changes in CAT expression (Figure 5, panel A).

Although several possible mechanisms such as impairment of NO production, activation of inflammatory pathways, telomere lenght and telomerase activity have been postulated as causes of endothelial dysfunction and vascular aging, increased production of ROS leading to accumulation of senescent cells is largely in charge of the regulation of vascular homeostasis [58]. In particular, increased oxidative stress has been suggested to contribute to the functional impairment of EPCs, by accelerating the rate of telomere shortening, inducing premature cells senescence, and increasing the formation of microparticles that, through a “feed-forward” mechanism, further increase ROS production. Oxidative stress is caused by the imbalance between the production of reactive oxygen species and a network of biological systems that form the antioxidant endogenous capability [59]. Previous studies from our group [37] and others [60], [61] demonstrated that human EPCs express high levels of antioxidant enzymes and are, as a result, more resistant to oxidative stress as compared to mature endothelial cells. For instance, in our experience, a significantly lower ROS production and a parallel marked increase in GPx-1 expression and activity were observed in human EPCs compared to HUVECs upon exposure to high constant D-glucose [37]. Consistently, Dernbach et al. [61] showed that EPCs exhibit a significantly lower basal ROS concentration as compared with HUVECs. Furthermore, addition of H_2_O_2_ resulted in a massive increase of ROS production and apoptosis in HUVECs, with only a minor increase in EPCs. The expression of CAT, GPx-1 and SOD was significantly higher in EPCs as compared with HUVECs. Thus, EPCs from healthy subjects seem well equipped with antioxidative defenses effective in providing resistance against oxidative insult [37], [61].

Resveratrol protects endothelial cells against oxidized low-density lipoprotein (oxLDL)-induced apoptosis [62]. Consistently, we have recently reported that LG has protective effects on human microvascular endothelial cells exposed to ox-LDL through reduction of oxidative/inflammatory processes [63]. Here, we highlight that LG and LJ protect EPCs against oxidative injury induced by H_2_O_2_. Cell viability, adhesiveness and senescence were significantly impaired under H_2_O_2_ stress (Figures 1–3, panels B); ROS production increased by about 60% (Figure 4, panel B); antioxidant protein expression showed eterogenous behaviour with increasing GPx-1 and SOD2, unchanging HO-1 and decreasing CAT (Figure 5, panel B). Pre-treatment of EPCs with LG or LJ at both 0.35 and 0.7 mg/ml concentrations protected the viability, adhesiveness and senescence level of EPCs exposed to H_2_O_2_, with all functional aspects almost completely restored by LJ (Figure 1–3, panels B). Both lysates showed only marginal effect at 1.4 mg/ml concentration. ROS production was reduced by both lysates at different concentrations, with an higher efficacy of 0.7 mg/ml LG (Figure 4, panel B). LG increased both SOD2 and HO-1 expression while LJ strongly improved SOD2 expression; finally no significant changes were observed for GPx-1 and CAT.

These results are consistent with an extensive literature showing that H_2_O_2_ induces oxidative damage, loss of cell viability, senescence and apoptosis of EPCs even in presence of a complex activation of the endogenous antioxidant defenses [43]. Here, we report for the first time that the enhanced activation of these systems might contribute to explain the ability of lysates to restore viability, adhesiveness and senescence of EPCs exposed to H_2_O_2_. Beneficial effects of EPCs functionality have been reported by treatment with several nutraceutical compounds. Extracts from Ginkgo biloba (ginkgolide B) protected EPCs from H_2_O_2_-induced cell death with involvement of Akt/endothelial NO synthase and MAPK/P38 signaling pathways [64]. Furthermore, Ginkgo biloba extract significantly improved SOD activity and decreased apoptosis in a dose-dependent manner in EPCs from diabetic subjects [65]. Also Salidroside significantly abrogated H_2_O_2_-induced EPCs apoptosis suppressing the H_2_O_2_-induced production of intracellular ROS [55]. Pre-treatment with Ecklonia cava-derived antioxidant dieckol suppressed the H_2_O_2_-induced ROS increase and drastically reduced the ratios of apoptotic EPCs [66]. Also some drugs exert cytoprotective effects in conditions of oxidative stress. One for all, trimetazidine, an anti-ischemic metabolic agent, protected the proliferation, adhesion, migration, and apoptosis of EPCs against H_2_O_2_ by an increase in both eNOS and SOD activities [67].

Nrf-2 is an essential transcription factor that plays a crucial role in cellular defense against oxidative stress. Nrf-2 serves as a “master regulator” of cell survival through the coordinated induction of phase II and antioxidant defense enzymes to counteract redox signaling events. In basal redox states, Nrf-2 is kept inactive into cytosol; otherwise, Nrf-2 translocates into the nucleus where activates ARE-containing genes encoding for antioxidant proteins. Expression of Nrf-2 in human aortic endothelial cells (HAECs) resulted in a marked increase in ARE-driven transcriptional activity and protected HAECs from H_2_O_2_-mediated cytotoxicity through upregulation of HO-1 and GPx-1 [68], [69]. Furthermore, hypoxic preconditioning increased nuclear translocation and ARE binding of Nrf2 and upregulated the expression of the antioxidative enzymes HO-1 and SOD [70]. Genistein, a phytoestrogen that belongs to the category of isoflavones, increased SOD, CAT and glutathione (GSH) levels and attenuated the decrease of these antioxidants during oxidative stress; it promoted the nuclear translocation of Nrf2 in an endothelial cell line [71]. To the best of our knowledge, only one study demostrated the activation of the Nrf-2 pathway in EPCs by nutraceuticals. Oleuropin and oleacein, present in olive oil and olive leaves respectively, restore biological functions of EPCs impaired by angiotensin II by reducing percentage of senescent cells and improving migration, adhesion and tube formation. This effect was related to Nrf-2 activation and increased HO-1 expression [72].

In our study, nuclear Nrf-2 translocation was visualized by fluorescence microscopy. LG, and only slightly LJ, induced nuclear Nrf-2 translocation both in basal conditions (Figure 6, panel B) as well as after exposure to the oxidative challenge (Figure 6, panel D). On the contrary, H_2_O_2_-induced stress, per se, was unable to activate Nrf-2 (Figure 6, panel C). Consistently, in our experimental setting, LG increased HO-1 expression irrespective of the H_2_O_2_-induced oxidative stress, whereas both LJ and H_2_O_2_ did not affect HO-1 expression. Thus, LG is able to activate a protective response not only before but also in the presence of an oxidative insult. Both lysates are rich in polyphenols, mainly LG, which are known to modulate Nrf-2/ARE pathway. We suggest that lysates-released polyphenols can work as ROS scavengers and can modulate gene expression involved in endogenous antioxidant response. Lisosan G, seems to directly activate Nrf-2 translocation into the nucleus.

The main limitations of the present study include both the lack of pre-clinical *in vivo* data as well as the unavailability of gene knockdown experiments designed to further evaluate the role of antioxidant marker genes in the context of lysate-mediated EPCs protection. Furthermore, in order to confirm antioxidative response induction, also enzymes activity might be evaluated other than other targets of Nrf-2 after lysates treatment.

In conclusion, the present study demonstrates that grain and bean lysates, LG and LJ, respectively, enhance viability and adhesion capacity of human EPCs thus reducing their senescence both in basal conditions as well after exposure to H_2_O_2_-induced oxidative stress. Expression of HO-1 and GPx-1 was increased in EPCs treated with LG and LJ, respectively, while expression of SOD2 was increased by both. These effects on antioxidant enzymes expression have been observed both in basal conditions as well after exposure to H_2_O_2_, suggesting that H_2_O_2_-induced cytotoxicity is counteracted by lysates. Finally, these data identify the Nrf2/ARE pathway as an endogenous system for antioxidant protection and upregulation of the redox-sensitive genes after treatment with Lisosan G. The inhibition of EPCs senescence by lysates *in vitro* may improve the functional properties of EPCs in a way that might be useful for potential cell therapy.
